# Association of fine particulate matter (PM_2.5_) exposure and chronic kidney disease outcomes: a systematic review and meta-analysis

**DOI:** 10.1038/s41598-024-51554-1

**Published:** 2024-01-10

**Authors:** Wannasit Wathanavasin, Athiphat Banjongjit, Jeerath Phannajit, Somchai Eiam-Ong, Paweena Susantitaphong

**Affiliations:** 1 Nephrology Unit, Department of Medicine, Charoenkrung Pracharak Hospital, Bangkok Metropolitan Administration, Bangkok, Thailand; 2 Nephrology Unit, Department of Medicine, Vichaiyut Hospital, Bangkok, Thailand; 3 Division of Nephrology, Department of Medicine, Faculty of Medicine, Chulalongkorn University, Bangkok, 10330 Thailand; 4 Division of Clinical Epidemiology, Department of Medicine, Faculty of Medicine, Chulalongkorn University, Bangkok, 10330 Thailand; 5 Center of Excellence for Metabolic Bone Disease in CKD Patients, Faculty of Medicine, Chulalongkorn University, Bangkok, Thailand

**Keywords:** Kidney diseases, Environmental impact, Pollution remediation

## Abstract

Several studies have reported an increased risk of chronic kidney disease (CKD) outcomes after long-term exposure (more than 1 year) to particulate matter with an aerodynamic diameter of ≤ 2.5 µm (PM_2.5_). However, the conclusions remain inconsistent. Therefore, we conducted this meta-analysis to examine the association between long-term PM_2.5_ exposure and CKD outcomes. A literature search was conducted in PubMed, Scopus, Cochrane Central Register of Controlled trials, and Embase for relevant studies published until August 10, 2023. The main outcomes were incidence and prevalence of CKD as well as incidence of end-stage kidney disease (ESKD). The random-effect model meta‐analyses were used to estimate the risk of each outcome among studies. Twenty two studies were identified, including 14 cohort studies, and 8 cross-sectional studies, with a total of 7,967,388 participants. This meta-analysis revealed that each 10 μg/m^3^ increment in PM_2.5_ was significantly associated with increased risks of both incidence and prevalence of CKD [adjusted odds ratio (OR) 1.31 (95% confidence interval (CI) 1.24 to 1.40), adjusted OR 1.31 (95% CI 1.03 to 1.67), respectively]. In addition, the relationship with ESKD incidence is suggestive of increased risk but not conclusive (adjusted OR 1.16; 95% CI 1.00 to 1.36). The incidence and prevalence of CKD outcomes had a consistent association across all subgroups and adjustment variables. Our study observed an association between long-term PM_2.5_ exposure and the risks of CKD. However, more dedicated studies are required to show causation that warrants urgent action on PM_2.5_ to mitigate the global burden of CKD.

## Introduction

Chronic kidney disease (CKD) remains a critical global public health concern with a high disease burden. The World Health Organization (WHO) reported that kidney disease was one of the top ten leading causes of mortality worldwide in 2019^1^. The incidence and prevalence of CKD are still increasing worldwide, by approximately 30% over the last 30 years^2,3^. This growing number emphasizes the significance of identifying the risk factors of CKD in order to devise prevention measures. Besides the traditional risk factors such as age and metabolic disorders (e.g., diabetes, hypertension, and obesity), several recent studies^4,5^ have suggested that environmental factors such as air pollution may play important roles in the disease process.

As a result of the rapid development of industrialization and urbanization, air pollution consequently becomes one of the major public health issues^6^ and has been listed as one of the most important contributors to the global burden of disease^7^. Air pollution is caused by a complex mixture of thousands of pollutants, which involve solid and liquid particles in suspension and a diverse array of gaseous elements^8^. Among various air pollutants, the US Environmental Protection Agency (EPA) and the European Union (EU) have selected particulate matter (PM) as a representative air pollutant, particularly PM with an aerodynamic diameter of less than 2.5 µm (PM_2.5_), when evaluating the health hazards of air pollution ^9^. Because of its small size, high surface area to volume ratio, and strong adsorption, PM_2.5_, also called fine particles, is strongly linked to toxic health effects^10^. A large number of studies have demonstrated that long-term exposure to PM_2.5_, which is defined by the 2021 global air quality guidelines of the WHO as 1 year to several years of exposure^11^, has been associated with various non-communicable diseases, including cardiovascular diseases^12,13^, respiratory diseases^14,15^, and neurodegenerative diseases^16^. Nevertheless, the existing data on the relationship between PM_2.5_ exposure and the risk of CKD has been less conclusive when compared with the aforementioned non-communicable diseases.

Recently, there has been increasing evidence for an association between PM_2.5_ and CKD. Physiologically, the human kidney is a vulnerable target for exposure to toxic substances, including PM_2.5_, due to their highly vascularized structure that receives 20%-25% of the cardiac output^17^. The proposed toxicological mechanism by which PM_2.5_ causes damage to the kidney, leading to a decline in estimated glomerular filtration rate (eGFR) and the development of CKD, is very complex. Most of the possible molecular pathways have been linked to an increase in pro-inflammatory cytokines, oxidative stress, and activation of the renin–angiotensin–aldosterone system and bradykinin cascade, causing DNA damage, autophagy, and eventually tissue fibrosis^18^. However, the recently reported results were inconsistent. To fill the research gap, this systematic review and meta-analysis aimed to explore whether long-term exposure to PM_2.5_ relates to adverse renal outcomes, including the risk of incidence and prevalence of CKD as well as the incidence of end-stage kidney disease (ESKD).

## Methods

We conducted this meta-analysis in accordance with the 2020 Preferred Reporting Items for Systematic Reviews and Meta-Analyses (PRISMA) guidelines for reporting interventions, along with a pre-registered protocol in the PROSPERO database (registration number CRD 42023457629).

### Searching strategy

Based on existing literature, a systematic search was implemented to search the literature on the relevance between PM_2.5 _and the CKD or ESKD outcomes. Our search encompassed the PubMed, Scopus, Cochrane Central Register of Controlled Trials (CENTRAL), and Embase databases up until August 10th, 2023, to identify relevant articles. The inception date of the search strategy was June 6th, 2023. The search terms utilized were (“particulate matter 2.5” OR “PM2.5”) AND (“kidney”[Mesh] OR kidney[tiab] OR “renal”[Mesh] OR renal[tiab]) in PubMed, and (“particulate matter 2.5” OR “PM2.5”) AND (kidney OR renal) in Scopus, CENTRAL, and Embase. Language restrictions were not imposed during the search process.

### Inclusion and exclusion criteria

The inclusion criteria of this meta-analysis comprised five points: (1) study subjects had to be adults (≥ 18 years); (2) studies had to examine long-term exposure (≥ 1 year) to fine particulate matter with an aerodynamic diameter of ≤ 2.5 μm (PM_2.5_); (3) only observational studies, including cross-sectional and cohort studies, were accepted; (4) the outcomes had to conclude the term “chronic kidney disease” or “end-stage kidney disease” explicitly for investigation with clinical assessments (such as diagnosed by physician, using the International Classification of Disease (ICD) code, or the Kidney Disease: Improving Global Outcomes (KDIGO) guideline); (5) studies reported the effect estimates (odds ratio; OR, and hazard ratio; HR) and their 95% confidence intervals (95% CIs) of clinical outcomes with per 10 μg/m^3^ increment exposure PM_2.5 _concentrations were available, or sufficient data could be used to convert these results. The exclusion criteria comprised three points: (1) reviews, meta-analyses, and responses to letters; (2) studies involving non-human species; and (3) the study reporting only specific chemical components of PM_2.5_-related adverse renal outcomes.

### Data extraction

The assessment of titles and abstracts for each record obtained, as well as the examination of full-text reports, was carried out independently by AB and WW. Whenever a discrepancy arose between the two reviewers, resolution was achieved through discussion involving the third author (PS). If multiple reports originated from the same cohort, the report with the largest sample size was selected. Subsequent data were extracted from each report, including the first author, year of publication, sampling period, study design type, research country, participant numbers, gender and age of participants, presence of diabetes and hypertension, mean body mass index, smoking habits, exposure assessment details, air pollutant data source, outcome and its assessment details, mean level of PM_2.5 _exposure, duration of follow-up, and risk of bias score.

### Assessments of quality and risk of bias

The evaluation of bias was conducted using the Newcastle–Ottawa Scale (NOS) for cohort studies and the modified NOS for cross-sectional studies^19,20^. The NOS encompasses a set of inquiries aimed at assessing the selection of study participants, the comparability of the population, and the outcomes. For cohort studies, the NOS was converted to adhere to AHRQ standards and categorized as follows: Good quality (3 or 4 stars in the selection domain AND 1 or 2 stars in the comparability domain AND 2 or 3 stars in the outcome/ exposure domain), Fair quality (2 stars in the selection domain AND 1 or 2 stars in the comparability domain AND 2 or 3 stars in the outcome/exposure domain), Poor quality (0 or 1 star in the selection domain OR 0 stars in the comparability domain OR 0 or 1 stars in the outcome/exposure domain). The adapted NOS for cross-sectional studies was designed with a maximum score of 10 points. Studies receiving 9–10 points were classified as very good, those with 7–8 points as good, those with 5–6 points as satisfactory, and those with 0–4 points as unsatisfactory. Utilizing these assessment criteria, both AB and WW conducted evaluations of the quality of each included article. Instances of differing opinions were resolved through consultation with a third author (PS).

### Statistical analysis

We conducted meta-analysis to extract combined effect estimates for the association of long-term PM_2.5_ exposure to CKD outcomes. The outcomes of the systematic review were classified into three categories: CKD prevalence, CKD incidence, and ESKD incidence. In each study, we extracted the adjusted effect estimates for every outcome, considering a more robust control for confounding variables. Within a subset of studies featuring analyzable and comparable data, expressing results as a standardized increment in PM_2.5_ concentration (μg/m^3^), the results were quantitatively synthesized. Odd ratios (ORs) were used as measurements of effect estimates across the included studies. If individual studies reported hazard ratios (HRs), we first converted these ratios into ORs using the method described by Shor et al.^21^ prior to calculating the pooled result. Random-effects model meta-analyses were performed to calculate pooled ORs for binary variables (i.e., presence versus absence of CKD outcomes) from multivariate analysis. Since the PM_2.5 _increment scales used to calculate the OR value in each study are inconsistent, which cause the effect values lack uniformity and cannot be combined. To circumvent this, we standardized the effect estimates (ORs and 95%CI) by pooling them based on a uniform per 10 μg/m^3^ increase in PM_2.5 _concentration. The standardized OR value for each article was calculated by using the formula as follows:

$${\text{Beta}} = \ln \;({\text{OR}}_{{({\text{original}})}} )$$

$${\text{Beta per }}10\;\upmu {\text{g}}/{\text{m}}^{3} \;{\text{increment}} = \ln \;({\text{OR}}_{{({\text{original}})}} ) \times \frac{10}{{Increment \left( {original} \right)}}$$

$${\text{OR}}_{{({\text{standardized}})}} = {\text{EXP}}\;({\text{Beta per }}10\;\upmu {\text{g}}/{\text{m}}^{3} \;{\text{increment}})$$

All pooled estimates were displayed with 95% CI. The presence of heterogeneity among the effect sizes of individual studies was assessed through the Cochrane's Q test and the I^2^ index. I^2^ values of 25%, 50%, and 75% or higher represent a low, moderate and high degree of heterogeneity, respectively. To explore sources of heterogeneity, we performed subgroup meta-analyses according to continents (Asia, Europe, or North America), sampling period (before 2013 or after 2013), study participants (< 10,000, 10,000–100,000, or > 100,000), mean PM2.5 level (< 25 μg/m^3^, or ≥ 25 μg/m^3^), based on WHO defining concentrations exceeding 25 μg/m^3^ as very high), pollutant data source (monitoring stations, predictive model, or machine learning), eGFR formula (Chronic Kidney Disease-Epidemiology Collaboration (CKD-EPI) equation or the Modification of Diet in Renal Disease (MDRD) study equation), and exposure periods (< 10 years, or ≥ 10 years). To graphically represent this heterogeneity among the included studies, a forest plot was employed. Publication bias was assessed formally using Funnel plots and the Egger test. All of these analyses were carried out using Comprehensive Meta-Analysis version 2.0 (www.meta-analysis.com, accessed on August 20, 2023; Biostat, Englewood, NJ, USA).

## Results

### Summary of included studies

**Figure 1:**
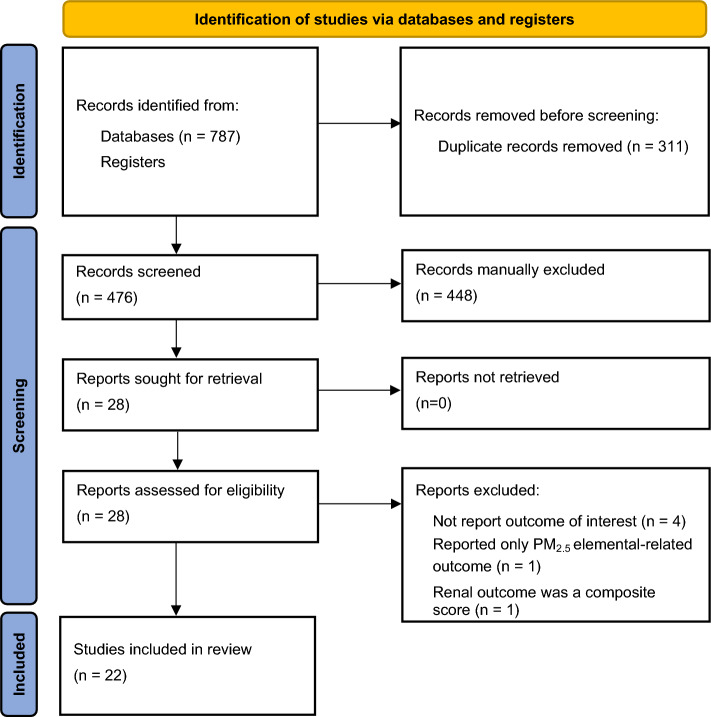
PRISMA 2020 flow diagram.

A total of 787 potentially relevant articles were initially identified through the database search. Following the removal of 311 duplicated articles, 476 article titles and abstracts underwent screening based on the inclusion and exclusion criteria, resulting in the identification of 28 full-text publications that underwent subsequent evaluation. After the full-text screening process, six articles were excluded (the reasons for exclusion are detailed in Fig. 1). Finally, a total of 22 articles were included for the systematic review and meta-analysis (Fig. 1).

The included articles were published from 2016 to 2023. The detailed characteristics and specific effect data of each article are listed in Table 1. Among the 22 selected articles, eight were cross-sectional studies, and the remaining fourteen were cohort studies. The study’s sample sizes were in the range of 2,083 to 2,938,653, with a total of 7,967,388 samples included in the meta-analysis. Three studies were conducted in North America (all in the USA)^22–24^, five in European countries (Sweden, the United Kingdom)^25–29^, and fourteen in Asia countries (China, Korea, Taiwan)^30–43^. These studies yielded a total sample of three continents (Asia: 48.6%, North America: 32.6%, and Europe: 18.8%). Of the included samples, the mean age was 53.5 years; 51.4% were male; 7.9%, and 39% had diabetes mellitus and hypertension as comorbidities, respectively; and 33.3% had a history of ever smoking.

**Table 1:** Characteristics of the studies included in the systematic review.

References	Sampling period	Study design	Country	Participants no	Mean Age	Men (%)	DM (%)	HT (%)	Mean BMI	Smoking (%)	Exposure assessment	Technique category	Outcome	Outcome assessment	Mean PM2.5 level (ug/m3) (SD or IQR)	F/U time (year)	Risk of bias score
Yang et al.^30^	2007–2009	cross-sectional	Taiwan	21,656	53.65	33.1	7.2	33.2	24.35	18.4	Land use regression model	Predictive model	Prevalent CKD	eGFR (CKD-EPI Taiwan) < 60	26.64 (5.67)	NA	8
Bowe et al.^22^	2003–2004	prospective cohort	US	2,482,737	62.46	95.19	27.8	67.26	NA	46.48	Annual monitor, modeled data	Predictive model	Incident CKD, ESKD	eGFR (CKD-EPI) < 60	11.8	8.52	8
Chen et al.^31^	2009	cross-sectional	Taiwan	8497	74.2	51	17.5	62.8	24.3	7.1	Land use regression model	Predictive model	Prevalent CKD	eGFR (CKD-EPI Taiwan) < 60	24.3 (3.9)	1	8
Lin et al.^32^	2000	prospective cohort	Taiwan	161,970	40.3	43.8	10.1	29.1	NA	NA	Monitoring stations nearby from the TAQMD	Montioring stations	Incident CKD, ESKD	ICD 9 codes: 580, 582, 583 (CKD), 585 (ESRD)	33.3	11.9	9
Blum et al.^23^	1987–2016	prospective cohort	US	10,997	63	44	16.5	47.5	28.8	58.7	Spatiotemporal generalized additive model	Predictive model	Incident CKD	eGFR (CKD-EPI) < 60 ml/min per 1.73 m2 with > 25% eGFR decline relative to baseline	12.85	17.7	8
Li et al.^33^	2009–2010	cross-sectional	China	47,204	49.6	42.7	7.4	35.2	23.9	23.5	Spatiotemporal model (monitoring and satellite data)	Predictive model	Prevalent CKD	eGFR (MDRD) < 60 or albuminuria, UACR (immunoturbidimetrics) > = 30	57.4 (15.6)	NA	9
Bo et al.^34^	2001–2016	prospective cohort	Taiwan	163,197	38.4	50.4	3.3	12.7	23	24.9	Spatiotemporal model (monitoring and satellite data)	Predictive model	Incident CKD	eGFR (MDRD) < 60 or self-reported physician	26.7 (7.7)	5.1	8
Ghazi et al.^24^	2012–2014	prospective cohort	US	100,894	48	46	9	30	NA	39	EPA downscaler model	Predictive model	Incident CKD	eGFR (CKD-EPI) < 60	10.1 (0.5)	8.5	9
Zeng et al.^35^	2001–2016	prospective cohort	Taiwan	104,092	38.4	51.9	3.2	12.3	22.9	24.9	Spatiotemporal model (monitoring and satellite data)	Predictive model	Incident CKD	eGFR (MDRD) < 60	26.8 (7.8)	6.7	8
Liang et al.^36^	2007–2010	cross-sectional	China	47,086	49.6	42.7	23.3	34.1	23.9	23.6	Satellite remote sensing inversion data, spatiotemporal model	Predictive model	Prevalent CKD	eGFR < 60 or urine protein creatinine > 30 mg/g	46.4 (15.3)	3	8
Xu et al.^25^	1991–1996	prospective cohort	Sweden	30,396	58	40	5	28	25.8	62	Gaussian dispersion model (AERMOD)	Predictive model	Incident CKD	eGFR < 60 or GFR > 60 with albuminuria or structural change of kidney	11	19.5	8
Oh et al.^37^	2016–2018	cross-sectional	Korea	15,983	47.5	43.7	NA	NA	23.96	39.9	Monitoring stations	Monitoring stations	Prevalent CKD	eGFR (CKD-EPI) < 60	24.69 (2.75)	NA	8
Duan et al.^38^	2005–2017	prospective cohort	China	72,425	38	58.3	6.8	27.9	23.4	33.2	China High Air Pollutants dataset	Machine learning	Incident CKD	eGFR (MDRD) < 60 or self-reported physician	68.6	3.79	8
Li et al.^26^	2006–2010	prospective cohort	UK	396,014	55.87	44.2	4.2	15.8	27.2	9.9	Land use regression model	Predictive model	Incident CKD	eGFR (CKD-EPI) < 60 or albuminuria ≥ 3 mg/mmol	10 (1.1)	11.7	8
Li et al.^39^	2018–2019	cross-sectional	China	80,225	51.8	39.6	NA	NA	NA	25.1	Satellite remote sensing data	Predictive model	Prevalent CKD	eGFR (CKD-EPI) < 60	40.7	NA	8
Wang et al.^27^	2006–2010	prospective cohort	UK	458,968	56.5	45.6	NA	47.2	24.5	10.4	Land use regression model	Predictive model	Incident CKD	ICD 10 codes (CKD-EPI)	10	11.7	7
Li et al.^40^	2017	cross-sectional	China	199,635	70.9	44.1	27.9	61	NA	15.4	China High Air Pollutants dataset	Predictive model	Prevalent CKD	eGFR (CKD-EPI) < 60, urine dipstick	28.2 (3.89)	NA	7
Liu et al.^41^	2013–2018	retrospective cohort	China	2082	46.9	60.7	11.4	29.9	NA	26.7	Land use regression model	Predictive model	Incident CKD	eGFR (CKD-EPI) < 60	NA	1	8
Wu et al.^28^	2006–2010	prospective cohort	UK	162,334	53.99	41.6	0	0	NA	42.9	Land use regression model	Predictive model	Incident CKD	eGFR (CKD-EPI) < 60 or ICD 10 codes	9.97	11.7	9
Zhang et al.^42^	2012–2017	cross-sectional	China	2,938,653	45	58.5	6.8	23.2	24.2	20.7	AOD data and the GEOS-Chem chemical transport model	Predictive model	Prevalent CKD	eGFR (MDRD) < 60	78.7 (22.5)	1	8
Wen et al.^43^	2017–2019	prospective cohort	China	8996	50.97	57.9	NA	NA	25	41.45	Tracking Air Pollution in China (TAP) database	Machine learning	Incident CKD	eGFR (MDRD) < 60	71.25 (8.61)	2.01	8
Li et al.^29^	2006–2010	prospective cohort	UK	453,347	59	45.9	5.26	26.8	26.75	95.5	Land use regression model	Predictive model	Incident CKD, ESKD	ICD 9 and ICD 10 code (CKD-EPI)	NA	11.87	8

*AERMOD* AMS/EPA regulatory model, *AOD* aerosol optical depth, *CKD* chronic kidney disease, *eGFR* estimated glomerular filtration rate, *EPA* environmental protection agency, *CKD-EPI* chronic kidney disease-epidemiology collaboration equation, *ICD* the international classification of disease, *MDRD* modification of diet in renal disease study equation, *TAQMD* Taiwan air quality monitoring network, *ESKD* end stage kidney disease, *UK* United Kingdom, *US* United States.

### Outcome assessment and exposure characteristics

The definition of CKD outcome among the included studies was consistently defined according to the KDIGO guidelines^44^. Specifically, the outcome was characterized by an eGFR lower than 60 mL/min per 1.73 m^2^, as predominantly determined by the CKD-EPI equation (n = 12; 57%) or MDRD equation (n = 6; 28.6%). Conversely, the ESKD outcome was primarily relied on the utilization of ICD codes. Of the included studies, the incidence of CKD was the most reported outcome (in 13 studies), followed by the prevalence of CKD (in 8 studies) and the incidence of ESKD (in 3 studies). Among the cohort studies, the reported incidences of CKD and ESKD ranged from 1.14% over 11.9 years to 27.3% over 17.7 years and 0.19% over 11.9 years to 1.29% over 8.52 years, respectively. In cross-sectional studies, the observed prevalence of CKD varied between 1.3 and 27.8%.

In the included studies, there are three main methods for assessing levels of PM_2.5 _exposure. The first one is obtained directly average air pollutant monitoring measurements from monitoring stations^32,37^; the second one is to use built models to make predictions^22–31,33–37,39–42^; and the last one is to use a machine-learning model^38,43^. Furthermore, the period of sampling encompassed the years 1987 to 2019, and the period of exposure assessment also varied across the included studies.

### Methodological quality

Regarding the Newcastle–Ottawa Scale (NOS) for cohort studies and the NOS adapted for cross-sectional studies^19,20^, all cohort studies (n = 13; 100%) were considered to be of good quality. Likewise, all of the cross-sectional studies (n = 9; 100%) were considered of good or very good quality (scores of 7–8). (see Supplementary Tables S1 and S2).

#### Association between long-term exposure to PM2.5 and adverse kidney outcomes

##### CKD incidence

**Figure 2:**
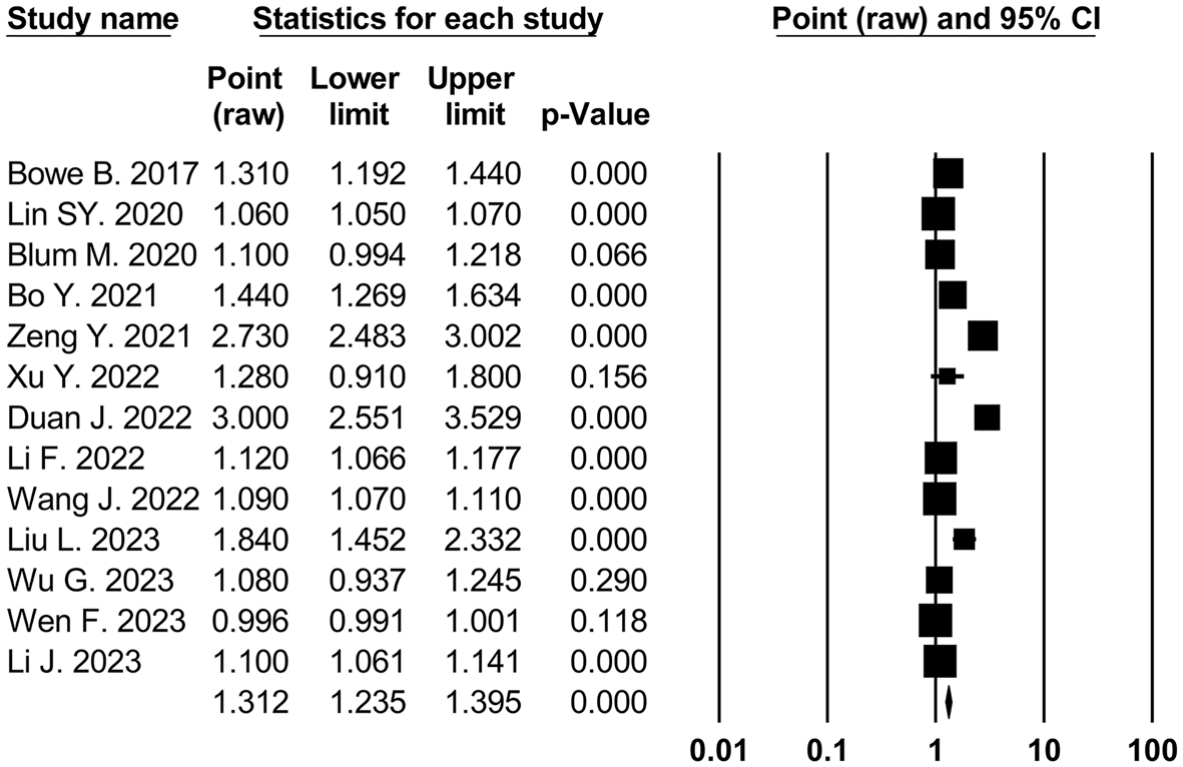
Forest plot displaying the pooled adjusted odds ratio of CKD incidence and long-term exposure to PM_2.5 _for increments of 10 μg/m^3^.

Thirteen cohort studies^22,23,25–29,32,34,35,38,41,43^ (3,663,102 participants) reported the association between long-term exposure to PM_2.5_ and incidence of CKD. The results of the analysis showed that every 10 μg/m^3^ increase in PM_2.5_ concentration of exposure was significantly associated with an increased risk of CKD incidence in both unadjusted analyses (OR 1.29, 95% CI 1.21 to 1.38;* p* < 0.001; 10 studies, 3,336,716 analyzed participants) (Table 2) and adjusted analyses (OR 1.31, 95% CI 1.24 to 1.40; *p* < 0.001; 13 studies, 3,663,102 analyzed participants) (Fig. 2, Table 2).

**Table 2:** Primary analysis examining the association between PM_2.5 _body exposure (per 10 μg/m^3^ increase) and risk of adverse kidney outcomes.

	No. of studies	No. of patients	Pooled odds ratio (95% CI)	*P*-values	Assessment of heterogeneity		Publication bias (Egger test)
					I^2^ index	*P*-value	*P*-value
CKD incidence							
Unadjusted	10	3,336,716	1.29 (1.21–1.38)	< 0.001	97.6%	< 0.001	0.003
Adjusted	13	3,663,102	1.31 (1.24–1.40)	< 0.001	98.7%	< 0.001	0.005
CKD prevalence							
Unadjusted	4	3,201,475	1.33 (1.28–1.37)	< 0.001	42%	0.159	0.92
Adjusted	6	3,112,218	1.31 (1.03–1.67)	0.026	97.6%	< 0.001	0.44
ESKD incidence							
Unadjusted	2	615,317	1.32 (0.85–2.04)	0.219	96.9%	< 0.001	NA
Adjusted	3	3,098,054	1.16 (1.00–1.36)	0.058	93%	< 0.001	0.36

*CKD* chronic kidney disease, *ESKD* end-stage kidney disease.

##### CKD prevalence

**Figure 3:**
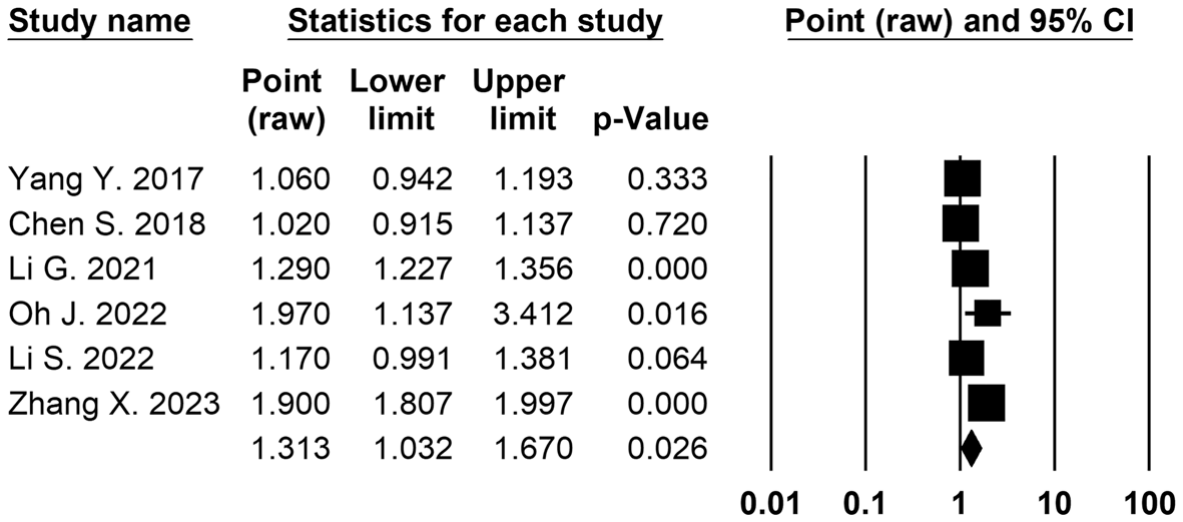
Forest plot displaying the pooled adjusted odds ratio of CKD prevalence and long-term exposure to PM_2.5 _for increments of 10 μg/m^3^.

Eight cross-sectional studies^30,31,33,36,37,39,40,42^ (3,359,057 participants) reported the association between long-term exposure to PM_2.5 _and CKD prevalence. It was found that every 10 μg/m^3^ increase in PM_2.5 _concentration of exposure was significantly associated with an increased risk of CKD prevalence in both unadjusted analyses (OR 1.33, 95% CI 1.28 to 1.37; *p* < 0.001; 4 studies, 3,201,475 analyzed participants) (Table 2) and adjusted analyses (OR 1.31, 95% CI 1.03 to 1.67; *p* = 0.026; 6 studies, 3,112,218 analyzed participants) (Fig. 3, Table 2).

##### ESKD incidence

**Figure 4:**
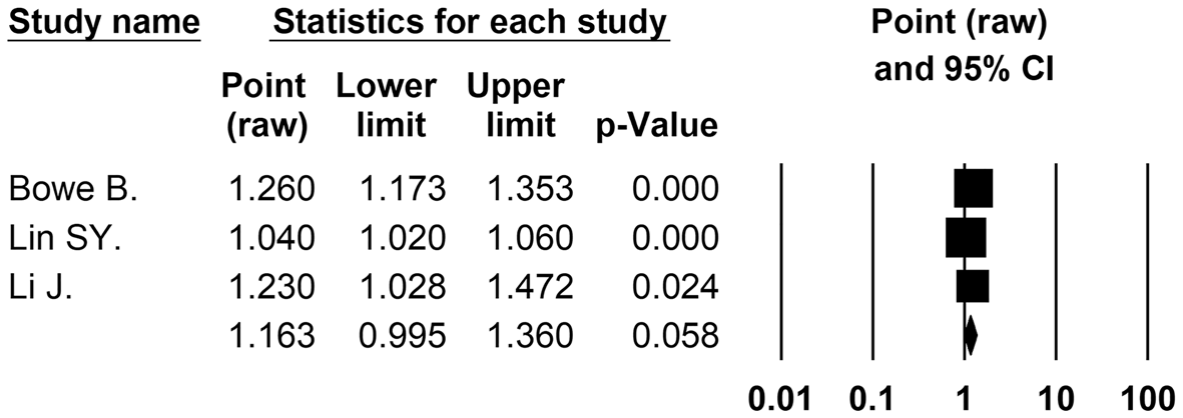
Forest plot displaying the pooled adjusted odds ratio of incidence ESKD and long-term exposure to PM_2.5 _for increments of 10 μg/m^3^.

Three cohort studies^22,29,32^ (3,098,054 participants) reported the association between long-term exposure to PM_2.5 _and incidence of ESKD. The result showed that the combined unadjusted and adjusted ORs of ESKD incidence in the meta-analyses were 1.32 (95% CI 0.85 to 2.04; *p* = 0.219; 2 studies, 615,317 analyzed participants) (Table 2) and 1.16 (95% CI 1.00 to 1.36; *p* = 0.058; 3 studies, 3,098,054 analyzed participants) (Fig. 4, Table 2) per 10 μg/m^3^ increase in PM_2.5 _concentration of exposure, respectively. Owing to the small number of included studies, we did not perform heterogeneity tests (such as subgroup analysis) on this outcome.

#### Investigations of heterogeneity

We found high heterogeneity in the estimated association among studies for all of the study outcomes (I^2^ = 98.7% for CKD incidence, I^2^ = 97.6% for CKD prevalence, and 93% for ESKD incidence). Therefore, we used the subgroup analyses to explore the potential confounding factors in the incidence and prevalence of CKD outcomes.

Tables 3 and 4 detail the results of subgroup analyses examining the association between PM_2.5_ body exposure (per 10 μg/m^3^ increase) and risk of incidence and prevalence of CKD, respectively, as stratified by continents (Asia, Europe, or North America), sampling period (before 2013 or after 2013), study participants (< 10,000, 10,000–100,000, or > 100,000), mean PM_2.5 _level (< 25 μg/m^3^, or ≥ 25 μg/m^3^), pollutant data source (monitoring stations, predictive model, or machine learning), eGFR formula (CKD-EPI or MDRD), exposure periods (< 10 years, or ≥ 10 years), and eight adjustment variables (comorbidity, smoking status, household income, urbanization, educational level, physical activity, temperature, and humidity).

**Table 3:** Subgroup analyses examining the association between PM_2.5_ body exposure (per 10 μg/m^3^ increase) and risk of CKD incidence.

Subgroup analyses	No. of studies	No. of patients	Pooled adjusted odds ratio (95% CI)	*P*-values	Assessment of heterogeneity	
					I^2^ index (%)	*P*-value
Continent						
Asia	6	512,762	1.58 (1.43–1.74)	< 0.001	99.36	< 0.001
Europe	5	1,495,687	1.10 (1.08–1.11)	< 0.001	0	0.746
North America	2	1,654,653	1.20 (1.01–1.43)	0.035	83.55	0.014
Sampling period						
Before 2013	7	3,302,008	1.11 (1.07–1.14)	< 0.001	81.10	< 0.001
After 2013 (Included 2013)	6	361,094	1.69 (1.11–2.57)	0.014	99.25	< 0.001
Study participants						
< 10,000	2	11,078	1.34 (0.73–2.44)	0.343	96.11	< 0.001
10,000–100,000	3	111,878	1.62 (0.78–3.36)	0.194	98.11	< 0.001
> 100,000	8	3,540,146	1.28 (1.17–1.41)	< 0.001	98.34	< 0.001
Mean PM_2.5_ level (µg/m3)						
< 25	6	2,701,120	1.14 (1.08–1.20)	< 0.001	67.50	0.009
≥ 25	5	510,680	1.55 (1.40–1.72)	< 0.001	99.47	< 0.001
Pollutant data source						
Monitoring stations	1	161,970	1.06 (1.05–1.07)	< 0.001	0	1.00
Predictive model	10	3,419,711	1.34 (1.17–1.52)	< 0.001	97.69	< 0.001
Machine learning	2	81,421	1.72 (0.59–5.08)	0.324	99.44	< 0.001
GFR formula						
CKD-EPI	7	3,123,271	1.15(1.09–1.21)	< 0.001	81.62	< 0.001
MDRD	4	348,710	1.85 (1.00–3.43)	0.051	99.53	< 0.001
Exposure period (year)						
< 10	6	1,995,143	1.74 (1.15–2.64)	0.009	99.28	< 0.001
≥ 10	7	1,667,977	1.09 (1.06–1.11)	< 0.001	58.65	0.024
Adjusted co-morbidities						
No	6	2,113,063	1.09 (1.04–1.15)	< 0.001	97.89	< 0.001
Yes	7	1,550,039	1.63(1.18–2.26)	0.003	98.62	< 0.001
Adjusted smoking status						
No	1	1,644,351	1.06 (1.05–1.07)	< 0.001	0	1.00
Yes	12	2,018,751	1.38 (1.25–1.52)	< 0.001	98.66	< 0.001
Adjusted income						
No	7	2,185,488	1.70 (1.21–2.37)	0.002	98.60	< 0.001
Yes	6	1,477,614	1.07 (1.02–1.12)	0.004	97.62	< 0.001
Adjusted urbanization						
No	9	3,040,470	1.53 (1.28–1.84)	< 0.001	98.48	< 0.001
Yes	4	622,632	1.06 (1.00–1.11)	0.041	97.95	< 0.001
Adjusted educational level						
No	6	2,113,063	1.42 (1.12–1.79)	0.003	98.81	< 0.001
Yes	7	1,550,039	1.27 (1.16–1.38)	< 0.001	98.13	< 0.001
Adjusted atmospheric temperature						
No	8	2,581,502	1.13 (1.08–1.17)	< 0.001	86.46	< 0.001
Yes	5	1,081,600	1.66 (1.05–2.64)	0.030	99.38	< 0.001
Adjusted physical activity						
No	6	2,113,063	1.13 (1.08–1.18)	< 0.001	89.54	< 0.001
Yes	7	1,550,039	1.51 (1.16–1.99)	0.003	99.10	< 0.001
Adjusted humidity						
No	10	3,042,552	1.11 (1.07–1.16)	< 0.001	96.81	< 0.001
Yes	3	620,550	2.27 (1.46–3.55)	< 0.001	97.35	< 0.001

*CKD-EPI* chronic kidney disease-epidemiology collaboration equation, *MDRD* modification of diet in renal disease study equation.

**Table 4:** Subgroup analyses examining the association between PM_2.5_ body exposure (per 10 μg/m^3^ increase) and risk of CKD prevalence.

Subgroup analyses	No. of studies	No. of patients	Pooled adjusted odds ratio (95% CI)	*P*-values	Assessment of heterogeneity	
					I^2^ index (%)	*P*-value
Sampling period						
Before 2013	3	77,357	1.12 (0.95–1.33)	0.172	90.31	< 0.001
After 2013 (Included 2013)	3	3,034,861	1.60 (1.08–2.37)	0.020	93.36	< 0.001
Study participants						
< 10,000	1	21,656	1.02 (0.92–1.14)	0.72	0	1.00
10,000–100,000	4	151,909	1.21 (1.05–1.40)	0.007	75.54	0.007
> 100,000	1	2,938,653	1.90 (1.81–2.00)	< 0.001	0	1.00
Mean PM_2.5_ level (µg/m^3^)						
< 25	2	30,153	1.34 (0.71–2.53)	0.37	81.17	0.021
≥ 25	4	3,082,065	1.33 (1.01–1.75)	0.044	98.16	< 0.001
Pollutant data source						
Monitoring stations	1	21,656	1.97 (1.14–3.41)	0.016	0	1.00
Predictive model	5	3,090,562	1.26 (0.98–1.62)	0.076	98.07	< 0.001
GFR formula						
CKD-EPI	4	93,340	1.10 (0.98–1.24)	0.114	54.72	0.085
MDRD	2	3,018,878	1.57 (1.07–2.29)	0.021	99.13	< 0.001
Adjusted co-morbidities						
No	1	21,656	1.17 (0.99–1.38)	0.064	0	1.00
Yes	5	3,090,562	1.35 (1.03–1.76)	0.031	98.02	< 0.001
Adjusted smoking status						
No	1	21,656	1.97 (1.14–3.41)	0.016	0	1.00
Yes	5	3,090,562	1.26 (0.98–1.62)	0.076	98.07	< 0.001
Adjusted income						
No	3	77,357	1.27 (0.80, 2.02)	0.-305	73.57	0.023
Yes	3	3,034,861	1.28 (1.13–1.45)	< 0.001	43.98	0.168
Adjusted urbanization						
No	4	93,340	1.10 (0.98–1.24)	0.114	54.72	0.085
Yes	2	3,018,878	1.57 (1.07–2.29)	0.021	99.13	< 0.001
Adjusted educational level						
No	1	21,656	1.90 (1.81–2.00)	< 0.001	0	1.00
Yes	5	3,090,562	1.17 (1.02–1.34)	0.027	83.20	< 0.001
Adjusted atmospheric temperature						
No	4	93,340	1.17 (0.99–1.38)	0.065	87.29	< 0.001
Yes	2	3,018,878	1.50 (0.93–2.41)	0.094	96.67	< 0.001
Adjusted humidity						
No	4	93,340	1.17 (0.99–1.38)	0.065	87.29	< 0.001
Yes	2	3,018,878	1.50 (0.93–2.41)	0.094	96.67	< 0.001

*CKD-EPI* chronic kidney disease-epidemiology collaboration equation, *MDRD* modification of diet in renal disease study equation.

In brief, all subgroups and adjustment variables considered in both the meta-analyses for incidence and prevalence of CKD consistently demonstrated that long-term exposure to PM_2.5 _(per 10 μg/m^3^ increase) was positively correlated with an elevated risk of both CKD outcomes (*P* for interaction > 0.05) (Tables 3 and 4).

#### Assessment of publication bias and sensitivity analysis

**Figure 5:**
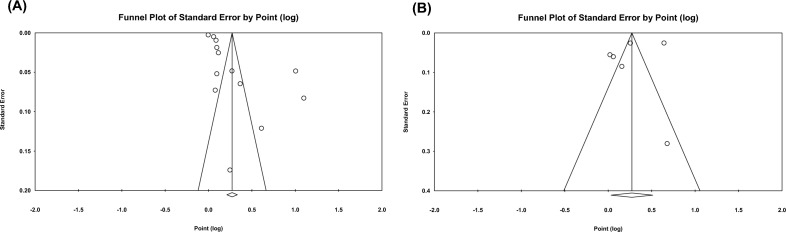
Funnel plot of individual studies displaying the standard error by the log odds ratio for (**A**) incident CKD (**B**) CKD prevalence outcome in long-term exposure to PM_2.5 _for increments of 10 μg/m^3^, *P* = 0.005, 0.44 by the Egger test, respectively.

The funnel plot for the outcome of both incidence and prevalence of CKD in the studies included in the meta-analysis was asymmetrical (Fig. 5A,B). The results of the Egger’s test suggested the presence of potential publication bias for CKD incidence outcome (*p* = 0.005), but not for CKD prevalence outcome (*p* = 0.44). The sensitivity analysis was conducted using the leave-one-out method (omitting one study at a time and recalculating the pooled effect estimate). The findings showed that the association between PM_2.5 _and incidence of CKD was generally stable and not dominated by any single study, which suggested that the results of the meta-analysis are substantially reliable.

## Discussion

In this systematic review and meta-analysis, we comprehensively evaluated and updated the existing epidemiologic evidence, including a total of nearly 8 million participants, on the association between long-term exposure to PM_2.5 _and adverse renal outcomes (incidence and prevalence of CKD as well as incidence of ESKD). Besides well-established risk factors for CKD, our analysis suggests that air pollution, particularly PM_2.5_, is identified as one of the emerging environmental risk factors, which has detrimental effects on kidney health. Of significance is the finding that long-term exposure to PM_2.5 _(per 10 μg/m^3^ increase) was associated with an elevated risk of CKD incidence (adjusted OR 1.31), CKD prevalence (adjusted OR 1.31). In addition, the relationship with ESKD incidence is suggestive of increased risk but not conclusive (adjusted OR 1.16; *p* = 0.058; when the follow-up duration extends beyond 10 years) (Figs. 2, 3, 4 and Table 2). High heterogeneity was noticed in the overall meta-analysis and most subgroup analyses, which may be attributed to the country development situation, continents, sampling period, sample size, mean PM_2.5 _level, pollutant data source, follow-up time, eGFR formula, temperature, and humidity. However, this finding might help explaining why CKD incidence and prevalence continued to increase on a global scale.

Table 5 illustrates the summary findings from four meta-analyses that reported the association between PM_2.5 _exposure and adverse kidney outcomes. Prior to the present study, there have been three meta-analyses on this topic, with one comprising 16 studies^45^, another encompassing 7 studies^46^, and the third including 13 studies^47^. Although several studies have performed meta-analyses on the impact of PM_2.5 _on CKD, none of these studies did a subgroup analysis to address the potential confounding factors that might affect this association. Of note, we found that specific subgroups (including continent, mean PM_2.5 _level, and sampling period) influenced the magnitude of this correlation, albeit in the same direction (Tables 3 and 4). Furthermore, two of these reports^45,47^ did not provide a clear definition of the CKD outcome, particularly in terms of distinguishing between incidence and prevalence of CKD. It is important to highlight that combining these outcomes together in the meta-analysis was not an appropriate approach. In the context of adverse renal outcomes, previous meta-analyses were limited to examining only CKD outcome; in comparison, our analysis broadened its scope by including an additional relevant outcome, which is the incidence of ESKD, with a longer observational period. Despite the consistent findings across these reports, all of which demonstrated a positive correlation between long-term exposures to PM_2.5 _(per 10 μg/m^3^ increase) and an elevated risk of CKD outcomes, the effect size was relatively small, ranging between 9 and 15% in terms of incremental risk. Moreover, one report by Wu et al.^45^ indicated borderline statistical significance in this context probably due to the limited number of the included studies (n = 4). Distinct from our systematic review and meta-analysis, the heightened magnitude of the effect size to 31% incremental risk of CKD incidence might be attributed to the incorporation of a larger sample size compared with the previous reports (Table 5).

**Table 5:** Summary of findings from 4 meta-analyses on the association between PM_2.5 _exposure and adverse kidney outcomes.

	Wu et al.^45^	Liu et al.^46^	Ye et al.^47^	The present meta-analysis
Year of publication	2019	2020	2021	2023
Data sources	Medline, EMBASE, The Cochrane Library	Pubmed, EMBASE, CINAHL, the Cochrane Library, Web of Science	Pubmed, EMBASE, Web of Science	Pubmed, Scopus, The Cochrane Library, EMBASE
Data search until	October 2019	May 2019	March 2020	August 2023
Exposure variable	PM_2.5_ exposure	PM_2.5_ exposure	PM_2.5_ exposure	PM_2.5_ exposure
Time of exposure	Long-term	Long-term	Long-term	Long-term
Comparator	Each 10 μg/m^3^ increment of PM_2.5_ exposure	Each 10 μg/m^3^ increment of PM_2.5_ exposure	Each 10 μg/m^3^ increment of PM_2.5_ exposure	Each 10 μg/m^3^ increment of PM_2.5_ exposure
Quality assessment tool	Cochrane Collaborations Risk of Bias tool and NOS scores	NOS scores and AHRQ	NOS scores and JBI-MAStARI	NOS scores and the modified NOS
Analytical approach	Random-effects model	Random-effects model	Random-effects model	Random-effects model
Total number of patients evaluated for PM_2.5_ exposure (total patients number)	3,991,321 (6,027,229)	3,777,576 (5,812,381)	4,006,149 (4,033,901)	7,967,388 (7,967,388)
Total number of studies	16	7	13	22
Cross-sectional	9	2	8	9
Case–control	0	0	0	0
Cohort	7	5	5	13
RCT	0	0	0	0
Proportion of studies included in the presented meta-analysis	4/16	2/7	4/13	22/22
Reported outcomes of meta-analysis	CKD GFR decline	Incident CKD	CKD	CKD incidence CKD prevalence ESKD incidence
Subgroup analysis	No	No	No	Yes
Pooled adjusted ORs (95%CI); number of studies analyzed	CKD 1.10 (1.00–1.21); n = 4	Incident CKD 1.09 (1.03–1.17); n = 5	CKD 1.15 (1.07–1.24); n = 10	CKD incidence 1.31 (1.24–1.40); n = 13 CKD prevalence 1.31 (1.03–1.67); n = 6 ESKD incidence 1.16 (1.00–1.36); n = 3

*AHRQ* agency for healthcare research and quality, *CI* confidence interval, *CKD* chronic kidney disease, *ESKD* end-stage kidney disease, *GFR* glomerular filtration rate, *JBI-MAStARI* Joanna Briggs institute meta-analysis of statistics asessment and review instrument, *NOS* Newcastle–Ottawa scale, *PM* particulate matter, *RCT* randomized control trial.

As a pollutant, PM_2.5 _is detrimental to public health due to its physical, chemical, and biological properties^48^, with the major components comprising elemental carbon, biological substances, inorganic components, organic components, and trace elements^49^. The sources can be either natural, such as coal burning and soil dust, or anthropogenic, such as vehicle traffic and industrial emissions^50^. Although the exact mechanisms through which PM_2.5 _induces kidney injury in humans remain unclear, it is hypothesized that PM_2.5 _primarily disrupts normal renal homeostasis via direct and indirect pathways^51^. Currently, the majority of the evidence explaining the direct pathways of kidney damage is derived from research conducted on animals. In summary, the identified mechanisms, mainly at the cellular level, encompass oxidative stress, inflammation leading to DNA damage, endoplasmic reticulum stress, apoptosis, and the development of renal fibrosis^52–54^. Furthermore, dysregulation of several systemic pathways such as angiotensin/ bradykinin systems, antioxidant, immune systems, and renal vascular activities has also been observed^55,56^. Apart from direct harmful effects, there is a growing body of evidence indicating that PM_2.5 _plays a substantial role in contributing to CKD through indirect pathways, primarily involving two major non-communicable diseases: hypertension^57,58^ and type 2 diabetes mellitus (T2DM)^59,60^, which serve as the principal drivers of CKD.

In subgroup analysis, we observed a notable association between PM_2.5 _and CKD incidence, particularly in the Asian region, despiteits smaller number of participants included in the analysis (Table 3). However, it is essential to note that the P for interaction > 0.05 when comparing Asian to other continents, indicating no statistically significant difference in the observed effects. In consistence with the World Health Organization (WHO)’s report^61^, less-developed regions, such as Asia and Africa, suffer PM_2.5 _exposures that are four to five times those of more-developed regions, including Europe and North America. The explanation behind this result lies in the rapid urbanization and economic growth observed in several Asian countries, which have led to a substantial increase in air pollution^62^.

Based on the “Air Quality Guideline” of the WHO^63^, an annual average of PM_2.5 _concentrations exceeding 25 μg/m^3^ is defined as a very high concentration, which can potentially have harmful effects on human health. The primary focus in terms of systemic diseases was on cardiovascular disease, respiratory disease, and lung cancers because of all linked to increased mortality risk^64^. The results from our analysis remained consistent in showing that individuals exposed to an average PM_2.5 _concentration higher than 25 μg/m^3^ had a greater risk of CKD outcomes in comparison to those with levels below 25 μg/m^3^ (Table 3). Of particular significance, these findings emphasize that kidney diseases should be recognized as another key public health concern related to the influence of PM_2.5_. Since the WHO designated PM_2.5_ as a Group 1 carcinogen in 2013, the global trend of PM_2.5_ concentration has gradually decreased over time due to its reduction policy. Therefore, we also conducted a subgroup analysis of the sampling period before and after 2013. Interestingly, the result showed that long-term PM_2.5_ exposure was more positively related to incident CKD and CKD prevalence in sampling periods after 2013 compared to before 2013 (Table 3). This could be clarified through the mechanism of renal injury, which involves a cumulative process requiring prolonged exposure to cause kidney damage.

Our systematic review has several strengths. This is the first systematic review and meta-analysis of observational studies that explores an association between long-term exposure to PM_2.5 _and adverse renal outcomes, particularly CKD incidence and CKD prevalence. We included reports that performed multivariable analyses to account for potential confounders of these associations. Furthermore, our search encompassed studies published until August 2023. It is worth noting that in the past few years, there has been a substantial increase in publications on this topic. This has resulted in a greater number of studies, a more diverse population, and more recent data, which reduces the possibility of residual confounding factors accounting for the observed association between PM_2.5 _and adverse renal outcomes. Admittedly, there are some important limitations that should be noted. First, our synthesis of the evidence was limited to observational studies, which implies that only correlation rather than causation can be demonstrated. Second, there was significant heterogeneity among the individual studies in terms of continents, sampling period, mean PM_2.5 _level, pollutant data source, eGFR formula, and meteorological parameters. Although we explored the potential sources of heterogeneity by conducting sensitivity analysis and subgroup analyses, the factors examined might account for only a partial explanation of the heterogeneity. Thus, most of the original studies did not control for important covariates, such as ethnicity/race, seasonal variations, the use of nephrotoxic agents, underlying cardiovascular disease and some unmeasured factors, which might also play a significant role in explaining the observed heterogeneity. Third, the definition of CKD also varied significantly among individual reports, and some cross-sectional studies conducted single-time tests, potentially impacting the accuracy of the diagnosis. This scenario was frequently observed in the setting of large-scale national surveys where the feasibility of repeated measurements was limited. Forth, we acknowledge the potential influence of publication bias, particularly affecting studies on CKD incidence, which may impact the robustness of our findings. Lastly, some effect estimates were not originally calculated but were converted, which might have biased the pooled result. Therefore, based on the aforementioned limitations, it is essential to interpret the results cautiously. There is an ongoing need for further high-quality prospective studies that control for significant confounding factors, identify specific populations or regions most vulnerable to the adverse effects of PM_2.5_, and define a robust outcome for accurate diagnosis in order to establish a causal relationship between PM_2.5_ exposure and CKD outcomes.

## Conclusion

In conclusion, our systematic review observed that long-term exposure to PM_2.5 _is associated with increased risks of CKD incidence and CKD prevalence. Hence, we emphasized that air pollution, particularly PM_2.5_, might be recognized as one of the emerging environmental CKD-related risk factors, which has detrimental effects on renal function. However, more dedicated studies are required to show causation that warrants urgent action on PM_2.5_ to mitigate the global burden of CKD.

### Supplementary Information

Supplementary Information.

## Data Availability

The datasets generated during and/or analyzed during the current study are available from the corresponding author on reasonable request.
