# Supplementary material for: Association of fine particulate matter (PM2.5) exposure and chronic kidney disease outcomes: a systematic review and meta-analysis
Source: Sci Rep. 2024 Jan 10;14:1048. doi: 10.1038/s41598-024-51554-1 (PMC10781728; doi:10.1038/s41598-024-51554-1)
Supplement: Supplementary file 1 — Supplementary Information. [file 41598_2024_51554_MOESM1_ESM.docx]

**Table S1** Risk of bias assessment using the Newcastle-Ottawa Scale adapted for cross-sectional studies. [1, 2]

|  | **Selection** | | | | **Comparability** | **Outcome** | | **Quality** |
| --- | --- | --- | --- | --- | --- | --- | --- | --- |
| First author (year) | Q1 | Q2 | Q3 | Q4 | Q1 | Q1 | Q2 |  |
| Li G. (2021)[3] | * | * | * | ** | * | ** | * | 9 (very good) |
| Zhang X. (2023)[4] | * | * |  | ** | * | ** | * | 8(good) |
| Liang Z. (2021)[5] | * | * |  | ** | * | ** | * | 8(good) |
| Yang Y. (2017)[6] | * | * |  | ** | * | ** | * | 8(good) |
| Chen S. (2018)[7] | * | * |  | ** | * | ** | * | 8(good) |
| Oh J. (2022)[8] | * | * |  | ** | * | ** | * | 8(good) |
| Li Y. (2023)[9] |  | * |  | ** | * | ** | * | 7(good) |
| Li S. (2022)[10] | * | * |  | ** | * | ** | * | 8(good) |

**Table S2** Risk of bias assessment using the Newcastle-Ottawa scale for cohort studies. [1, 2]

|  | **Selection** | | | | **Comparability** | **Outcome** | | | **Quality** |
| --- | --- | --- | --- | --- | --- | --- | --- | --- | --- |
| **First author (year)** | **Q1** | **Q2** | **Q3** | **Q4** | **Q1** | **Q1** | **Q2** | **Q3** |  |
| Duan J. (2022)[11] | * | * | * | * | ** | * | * |  | Good |
| Bo Y. (2021)[12] | * | * | * | * | ** | * | * |  | Good |
| Liu L. (2023)[13] | * | * | * | * | ** | * | * |  | Good |
| Zeng Y. (2021)[14] | * | * | * | * | ** | * | * |  | Good |
| Wen F. (2023)[15] | * | * | * | * | ** | * | * |  | Good |
| Li F. (2022)[16] | * | * | * | * | ** | * | * |  | Good |
| Blum M. (2020)[17] | * | * | * | * | ** | * | * |  | Good |
| Bowe B. (2018)[18] | * | * | * | * | ** | * | * |  | Good |
| Wang J. (2022)[19] |  | * | * | * | ** | * | * |  | Good |
| Xu Y. (2022)[20] | * | * | * | * | ** | * | * |  | Good |
| Wu G. (2023)[21] | * | * | * | * | ** | * | * | * | Good |
| Lin S. (2022)[22] | * | * | * | * | ** | * | * | * | Good |
| Ghazi L. (2022)[23] | * | * | * | * | ** | * | * | * | Good |
| Li J. (2023)[24] | * | * | * | * | ** | * | * |  | Good |

**References**

1. Wells GA, Shea B, O’Connell D, Peterson J, Welch V, Losos M, et al. The Newcastle-Ottawa Scale (NOS) for Assessing the Quality of Nonrandomised Studies in Meta-Analyses [cited 2022 06 Aug]. Available from: <https://www.ohri.ca/programs/clinical_epidemiology/oxford.asp>.

2. Herzog R, Alvarez-Pasquin MJ, Diaz C, Del Barrio JL, Estrada JM, Gil A. Are healthcare workers' intentions to vaccinate related to their knowledge, beliefs and attitudes? A systematic review. BMC Public Health. 2013;13:154.

3. Li G, Huang J, Wang J, Zhao M, Liu Y, Guo X, et al. Long-Term Exposure to Ambient PM(2.5) and Increased Risk of CKD Prevalence in China. J Am Soc Nephrol. 2021;32(2):448-58.

4. Zhang X, Tao J, Lei F, Sun T, Lin L, Huang X, et al. Association of the components of ambient fine particulate matter (PM(2.5)) and chronic kidney disease prevalence in China. J Environ Manage. 2023;339:117885.

5. Liang Z, Wang W, Wang Y, Ma L, Liang C, Li P, et al. Urbanization, ambient air pollution, and prevalence of chronic kidney disease: A nationwide cross-sectional study. Environ Int. 2021;156:106752.

6. Yang YR, Chen YM, Chen SY, Chan CC. Associations between Long-Term Particulate Matter Exposure and Adult Renal Function in the Taipei Metropolis. Environ Health Perspect. 2017;125(4):602-7.

7. Chen SY, Chu DC, Lee JH, Yang YR, Chan CC. Traffic-related air pollution associated with chronic kidney disease among elderly residents in Taipei City. Environ Pollut. 2018;234:838-45.

8. Oh J, Ye S, Kang DH, Ha E. Association between exposure to fine particulate matter and kidney function: Results from the Korea National Health and Nutrition Examination Survey. Environ Res. 2022;212(Pt A):113080.

9. Li Y, Yuan X, Wei J, Sun Y, Ni W, Zhang H, et al. Long-term exposure to ambient particulate matter and kidney function in older adults. Atmospheric Environment. 2023;295:119535.

10. Li S, Meng Q, Laba C, Guan H, Wang Z, Pan Y, et al. Associations between long-term exposure to ambient air pollution and renal function in Southwest China: The China Multi-Ethnic Cohort (CMEC) study. Ecotoxicol Environ Saf. 2022;242:113851.

11. Duan JW, Li YL, Li SX, Yang YP, Li F, Li Y, et al. Association of Long-term Ambient Fine Particulate Matter (PM(2.5)) and Incident CKD: A Prospective Cohort Study in China. Am J Kidney Dis. 2022;80(5):638-47 e1.

12. Bo Y, Brook JR, Lin C, Chang LY, Guo C, Zeng Y, et al. Reduced Ambient PM(2.5) Was Associated with a Decreased Risk of Chronic Kidney Disease: A Longitudinal Cohort Study. Environ Sci Technol. 2021;55(10):6876-83.

13. Liu L, Tian X, Zhao Y, Zhao Z, Luo L, Luo H, et al. Long-term exposure to PM(2.5) and PM(10) and chronic kidney disease: the Beijing Health Management Cohort, from 2013 to 2018. Environ Sci Pollut Res Int. 2023;30(7):17817-27.

14. Zeng Y, Lin C, Guo C, Bo Y, Chang LY, Lau AKH, et al. Combined effects of chronic PM(2.5) exposure and habitual exercise on renal function and chronic kidney disease: A longitudinal cohort study. Int J Hyg Environ Health. 2021;236:113791.

15. Wen F, Xie Y, Li B, Li P, Qi H, Zhang F, et al. Combined effects of ambient air pollution and PM(2.5) components on renal function and the potential mediation effects of metabolic risk factors in China. Ecotoxicol Environ Saf. 2023;259:115039.

16. Li FR, Zhu B, Liao J, Cheng Z, Jin C, Mo C, et al. Ambient Air Pollutants and Incident Microvascular Disease: A Cohort Study. Environ Sci Technol. 2022;56(12):8485-95.

17. Blum MF, Surapaneni A, Stewart JD, Liao D, Yanosky JD, Whitsel EA, et al. Particulate Matter and Albuminuria, Glomerular Filtration Rate, and Incident CKD. Clin J Am Soc Nephrol. 2020;15(3):311-9.

18. Bowe B, Xie Y, Li T, Yan Y, Xian H, Al-Aly Z. Particulate Matter Air Pollution and the Risk of Incident CKD and Progression to ESRD. J Am Soc Nephrol. 2018;29(1):218-30.

19. Wang J, Li D, Sun Y, Tian Y. Air pollutants, genetic factors, and risk of chronic kidney disease: Findings from the UK Biobank. Ecotoxicol Environ Saf. 2022;247:114219.

20. Xu Y, Andersson EM, Krage Carlsen H, Molnar P, Gustafsson S, Johannesson S, et al. Associations between long-term exposure to low-level air pollution and risk of chronic kidney disease-findings from the Malmo Diet and Cancer cohort. Environ Int. 2022;160:107085.

21. Wu G, Cai M, Wang C, Zou H, Wang X, Hua J, et al. Ambient air pollution and incidence, progression to multimorbidity and death of hypertension, diabetes, and chronic kidney disease: A national prospective cohort. Sci Total Environ. 2023;881:163406.

22. Lin SY, Ju SW, Lin CL, Hsu WH, Lin CC, Ting IW, et al. Air pollutants and subsequent risk of chronic kidney disease and end-stage renal disease: A population-based cohort study. Environ Pollut. 2020;261:114154.

23. Ghazi L, Drawz PE, Berman JD. The association between fine particulate matter (PM(2.5)) and chronic kidney disease using electronic health record data in urban Minnesota. J Expo Sci Environ Epidemiol. 2022;32(4):583-9.

24. Li J, Dai L, Deng X, Zhang J, Song C, Xu J, et al. Association between long-term exposure to low level air pollutants and incident end-stage kidney disease in the UK Biobank: A prospective cohort. Chemosphere. 2023;338:139470.
